# Performance of the non-invasive point-of-care device, EzeCheck, for haemoglobin assessment in adults and children in community and institutional care settings

**DOI:** 10.1371/journal.pdig.0000500

**Published:** 2024-05-08

**Authors:** Partha Pratim Das Mahapatra, Chaitali Roy, Komal Agarwal, Joy Banerjee, Sandeep Sharma

**Affiliations:** EzeRx Health Tech Pvt. Ltd, Bhubaneswar, Odisha, India; Brown University, UNITED STATES

## Abstract

Anaemia is a major public health problem, especially in resource constrained settings. Dependency on assessment of blood hemoglobin (Hgb) concentration impedes anemia detection, risk stratification and intervention. Thus, valid, frugal and scalable technologies are needed. EzeCheck is a noninvasive portable device developed in India for predicting hemoglobin levels in human beings aged 4 years and above using a finger-tip biosensor. In this assessment, we aimed to: (i) evaluate EzeCheck’s performance with an automated whole blood hemato-analyzer (Sysmex XN 1000) as the gold standard, and (ii) estimate EzeCheck’s agreement with Sahli’s method and HemoCue (Hb-301) in real-world primary and higher care facilities. Paired assessments were done at five sites across India i.e., Bhubaneshwar, Shimla, Solan and Mashobra and Ulhasnagar. Participants across all age groups (4 years and above) were assessed. We used a range of statistical tests to evaluate the performance of EzeCheck. It was found that EzeCheck performed well across age and gender categories with convincing validity, concordance, precision and accuracy, and acceptable bias. While comparing EzeCheck with Hemato-analyzer, no statistically significant systematic bias was found. However, EzeCheck showed significant systematic bias when compared to Sahli’s method and HemoCue. We concluded that EzeCheck could detect anemia (as per WHO Hgb cut-offs) in ‘real-world settings’ and ‘across age and gender categories’, with high sensitivity, specificity and accuracy, and can serve as a replacement to traditional methods of hemoglobin assessment. Further, for countries with higher prevalence of anemia where universal screening may be mandated, the positive predictive value of EzeCheck will be higher. The likelihood ratios also indicated that the device had moderate-to-good utility. EzeCheck is suitable for embedment into program and out-patient health care settings in resource constrained contexts as a spot-check hemoglobinometer.

## Author summary

Due to increasing rates of anaemia prevalence specially among women and children, need for the quick and effective healthcare technology arises. Anaemia adversely affects the mental health development in children, can lead to pregnancy complications, heart problems, weakness, etc., and could be life-threatening if the haemoglobin levels are too low and timely intervention and care is not performed. Use of non-invasive tools such as EzeCheck could help in achieving the timely management of the disease. To access the effectiveness of the device, a cross-sectional study was conducted at 5 different geographical regions of India. Results are promising and in concordance with our statement which highlights the use of convenient non-invasive methods for timely and early diagnosis for anaemia. For conducting mass level screening programmes particularly in rural areas, where healthcare facilities are limited, a quick, handy and effective method for determining haemoglobin levels, could help the government in disease diagnosis at a preliminary level and for determining effective treatment plans.

## Introduction

Anaemia is a major public health problem across several nations world-wide, and especially in resource constrained settings. Anemia leads to adverse health outcomes at the individual level, affects population across age groups and gender, and diminishes overall economic productivity of the nation. In response, countries have deployed mass iron-folate supplementation and deworming programs often targeted at high-risk groups i.e., pregnant women, adolescent girls and children. The success of these programs, however, have been quite inconsistent. Assessment of Hemoglobin (Hgb) concentration is widely considered for screening individuals for anemia [1,2]. One of the major hurdles in combating anemia effectively, however, is the dependency on assessment of hemoglobin (Hgb) concentration in the blood for anemia detection, risk stratification and intervention. It is crucial that the techniques or methods used to detect anemia within resource constrained program and healthcare ambits be valid, frugal and scalable [3].

The conventional method of Hgb measurement requires drawing venous blood by a nurse/phlebotomist and subsequent analysis in a laboratory by a technician or by an automated haemato-analyzer equipment [4]. Thus, laboratory-based Hgb testing usually requires venous blood draw, trained technicians and expensive equipment [5]. Although the method is deemed as accurate, it entails cost for transportation and processing, delay in reporting the results, biomedical waste generation, blood loss and discomfort to the patients [6–8]. These different factors have led towards the development of more readily usable point-of-care-devices for Hgb measurement. These devices have attempted to minimize the need of a venous blood tap by using a finger-tip prick capillary sample instead [9–10]. The sample thus collected is used to instant processing followed by an estimate of the Hgb concentration. These devices have been quite popular in field settings but require time-to-time recalibration; the reagent also should be protected from exposure to direct sunlight and extreme temperature conditions to give valid results. Subsequently, teams have explored the options for developing POC Hgb estimation devices that not just overcome the above inconsistencies but also obviate the need for needle prick and blood draw. Further, these non-invasive POC devices have also focused on the ease of use, portability, affordability in resource constrained settings, and on shortening the reporting time leading to improved patient satisfaction [5–8].

EzeCheck is a noninvasive portable device developed in India which can predict hemoglobin levels in human beings aged 4 years and above using a fingertip-based sensor, within a minute. The device is developed and manufactured by EzeRx Health Tech Private Limited, which is an Odisha based Med-Tech company. EzeRx aims to bring medical care closer and easier to the patient and their physicians by developing and manufacturing advanced medical devices and providing them with innovative solutions. The organization is awarded on numerous platforms for its innovation, most remarkably being awarded the Gold Award for healthcare by Infosys Foundation among many others.

The EzeCheck device has received recognition from the Department of Industrial Policy & Promotion and Startup Odisha for its innovative approach towards mankind. The device is IEC 60601, IEC 60601-1-2, ISO 13485 and CDSCO approved.

The device was developed with an idea to make non-invasive and affordable preventive healthcare devices to reach people in remote locations for early diagnosis of disease [11]. The device has been used in India in hospital and community settings. In this study, for the non-invasive EzeCheck point-of-care device for Hgb assessment, we aimed to: (i) evaluate its performance with an automated whole blood haemato-analyzer (Sysmex XN 1000) as the gold standard, and (ii) estimate its agreement with other popular methods of Hgb assessment i.e., the traditional Sahli’s (acid hematin) method and routinely-used portable invasive (finger-prick) hemoglobinometer HemoCue (Hb-301) in real-world primary and higher care facilities. By exploring the performance of the POC device, we hope potential users will be empowered to make informed choice about using the device at appropriate places.

## Methods

### Design and setting

With permission from local administrators and competent authorities, we assessed the Hgb of individuals with EzeCheck along with at least one more method (Hemocue Portable Photometer Hb-301, Haematology Analyzer, and Sahli’s Method; Box 1). This was done at five sites located across three states (Odisha, Maharashtra and Himachal Pradesh) in India. Thus, using a cross-sectional approach, paired assessments were done at Bhubaneshwar (State: Odisha in Eastern India) in July and August 2021, at Shimla, Solan and Mashobra (State: Himachal Pradesh in Northern India) in August and September 2022, and at Ulhasnagar (State: Maharashtra in Western India) in July 2022. All the assessments were done during the monsoon season.

**Box 1. Methods of Hemoglobin assessment used across the sites**

*EzeCheck*: EzeCheck is a non-invasive portable device which can predict the hemoglobin concentration in the human body, instantly, using spectrophotometry technique. The device has four main components: (a) a printed circuit board (PCB) with the sensor, (b) mobile application, (c) web application and (d) an Artificial Intelligence (AI) Algorithm. The sensor is placed on the pulp of the left-hand ring finger of the individual. The device then uses a cool white Light Emitting Diode (LED) light and projects it onto the ring finger. The reflection of the LED light that bounces from the anterior side of the finger is collected by EzeCheck, and sent to the mobile application. The application is connected to server which runs an AI algorithm to analyze the biomarkers present in the light signal and predict Hgb levels. The measurements are then sent back to the android application for display to the patient [11].

*Haemocue Portable Photometer Hb-301*: The Haemocue is a method of point-of-care measurement of Haemoglobin. It is a small, portable, and easy to use POC device used widely by healthcare providers worldwide. The blood sample obtained is either venepuncture or capillary blood by finger prick. It consists of disposable microcuvettes containing reagent and one time use designed photometer. Within the microcuvettes sodium deoxycholate haemolyses the red blood cells releasing haemoglobin. Sodium nitrite then converts the haemoglobin to methemoglobin which, together with sodium azide, gives azide methemoglobin. The absorbance of the sample is measured at two wavelengths (570 and 880 mm) in order to compensate for turbidity in the sample. The haemoglobin level is then calculated by the meter and displayed on the screen [12–14].

*Hematology Analyzer (Sysmex XN-1000)*: Hematology Analyzer is an automatic multi-parameter blood cell counter for in-vitro diagnostic use in clinical laboratories. It is a compact, fully automated hematology analyzer with simultaneous analysis of multiple parameters in whole blood mode and capillary blood mode. This instrument uses cyanide-free sodium lauryl sulphate (SLS) haemoglobin detection method. The result appears on the screen of the Information Processing Unit) IPU [15].

*Sahli’s Method*: Sahli’s method of Haemoglobin estimation is a non-invasive traditional method. The equipment consists of a comparator with a brown glass standard and Sahli’s graduated haemoglobin tube which is marked in percent and gram. In this method blood is mixed with an acid solution, the haemoglobin converts into the brown-coloured acid hematin. The acid hematin is then diluted with distilled water till the colour of the acid hematin matches that of the brown glass standard. The haemoglobin is then estimated by reading the value directly from the scale [16,17].

While the assessments were conducted in a tertiary-care hospital setting in Bhubaneshwar (Capital Hospital), Solan (Regional Hospital), Shimla (Kamla Nehru Hospital for Mother and Child) and in Ulhasnagar (Government Central Hospital), in Mashobra, these were done at the primary-care Community Health Centre (CHC).

### Study population

We included participants across all age groups starting from the age of four years and beyond. We did not include individuals under the age of four years in the assessments because in case of children under 4 years, the fingertip area is too thin to completely cover the finger bed region of the device, lack of which will result in light passage causing improper light absorption from the fingertip, ultimately leading to erroneous results.

We assessed consecutive individuals at these centres as per operational feasibility. We did not restrict individuals from participating in the assessment except that those who had not been advised Hgb estimation as part of the routine consultation, those with tar, soot or mehndi on the fingers, and those who declined a verbal consent were excluded.

### Data collection

Participants were assessed using the routinely available method for Hgb assessment at the respective site i.e. the automated Hematology Analyzer (Sysmex XN-1000) in Bhubaneshwar, Solan and Ulhasnagar, and Sahli’s method in Shimla and Mashobra, using venous blood samples. Individuals at Ulhasnagar were also tested using HemoCue on finger prick sample (capillary blood). Additionally, along with the routine methods, participants across the sites were also assessed for Hgb level using EzeCheck.

First, for the consenting individuals, we captured information on the age and gender of the individuals recorded in the forms routinely used across the sites; other variables were not available as the forms used across the sites were not standardized. This was entered in the mobile application to which the EzeCheck device was connected through Bluetooth. Once the entry was complete, the device was set to capture the signal from the reflection of the LED light for estimation of the Hgb levels. The EzeCheck sensor was placed on the pulp of the left hand ring finger of the participant. The device ran a series of signal processing techniques at the backend by the machine learning algorithm and generated a value for the haemoglobin on the mobile screen for that participant in about 30 seconds; the reading was archived in the mobile app, which could be easily viewed (in the form of pdf), whenever required in the future. Each of the participant was measured only once.

Immediately after being assessed using EzeCheck, the participant’s venous (for Hematology Analyzer and Sahli’s Method)/ capillary (for HemoCue) blood samples were collected for Hgb assessment using the alternative method(s).

The capillary blood samples for estimates through HemoCue were collected by a finger prick from the ring finger of the left hand. The finger prick was done after cleaning and massaging the finger to facilitate blood flow. For the Sahli’s Method and Hematology Analyzer, venous blood samples were collected from the left antecubital vein into vacutainer tubes containing ethylenediamine tetra acetic acid (EDTA). Alternative venous sites were chosen for sample collection if the preferred site (as mentioned above) was not suitable. All the tubes with blood samples from participants were sent to the pathology laboratory associated with the respective health facility and were analysed within 24 hours from the time of collection.

Reading from HemoCue was also recorded in real-time while those for Sahli’s method and the autoanalyzer was captured from the respective laboratories.

### Data management and analysis

The data of the participants and their tested Hgb levels were stored in the EzeCheck web application. The data was then exported in digital format and reported in MS Excel. Statistical analyses of the data were performed using STATA software (StataCorp, version 16.0) [18] and MS Excel.

We undertook descriptive analyses (frequency, proportion, and mean and standard deviation/ median with range, as appropriate) to provide a profile of the samples used.

*For assessing EzeCheck’s validity*: For comparing the performance of EzeCheck with Autoanalyzer as the gold standard, we categorised the Hgb readings with each method as ‘anaemia’ and ‘no anaemia’. We used the sex and age-specific WHO criteria [19] as cut-offs: children aged 4–11 years with Hgb <11.5 g/dL, adolescents aged 12–18 years with Hgb <12 g/dL, men aged more than 18 years with Hgb < 13.0 g/dL and women more than 18 years with Hgb <12.0 g/dL were categorized as ‘anaemia’ and the rest as ‘non-anaemia’. We undertook an assessment of EzeCheck’s sensitivity (Sn), specificity (Sp), positive and negative predictive values (PPV and NPV respectively), and positive and negative likelihood ratio (LR+ and LR- respectively). We used the *diagtest* command in STATA with further computation, as needed. For estimating the accuracy of EzeCheck, we computed the area under the receiver operating characteristic (ROC) curve with Autoanalyzer as dichotomous and EzeCheck as a continuous variable. To identify the optimal cut-off for EzeCheck readings to detect anaemia/ no anaemia, we calculated Youden Index (1- (1-Sp)) and reported the cut-off along with the corresponding sensitivity and specificity.

*For assessing EzeCheck’s agreement with other methods*: For assessing the agreement between EzeCheck with HemoCue and Sahli’s Method (i.e. objective 2), we undertook Passing-Bablok regression analyses (for reporting constant bias (intercept; deemed not statistically significant if 95% confidence interval included the value 0), proportional bias (slope; deemed not statistically significant if 95% confidence interval included the value 1), random bias (residual standard deviation; reported as % mean with % standard deviation), and linear relationship between the tests (using cusum test)), Bland-Altman analyses (for reporting bias with upper and lower levels of agreement with a mean difference of 1.0 gm/dl as the acceptable limit)[20], computed Lin’s concordance correlation coefficient of absolute agreement (r_ccc_; deemed as ‘good’ if r_ccc_>0.8) along with accuracy (bias corrected factor, C_b_) and precision (Pearson’s *r*), and calculated Spearman-Brown split-half correlation coefficient ((*sbsc;* we used the formula sbsc = 2*rho/ (1+rho)). For *sbsc*, we deemed a value of ≥0.99 as ‘perfect’, 0.95-<0.99 as ‘substantial’, 0.90-<0.95 as ‘moderate’, and <0.90 as ‘poor’ agreement. We checked for difference between the readings of EzeCheck with either method using Student’s t-test for paired samples. All the variables were treated as continuous, and the tests were undertaken after verifying for normality of distribution using scatter plots, Q-Q plots and Shapiro-Wilk analysis. We assumed statistical significance if p-value was less than 0.05.

### Ethics

All potential participants across the sites were explained about EzeCheck before assessment. Verbal informed consent was obtained from all adult participants before measuring their haemoglobin using EzeCheck and other comparator method(s). If a participant was aged more than 12 years but less than 18 years, assent was taken from the individual along with consent from either parent or guardian. For participants under the age of 12 years, surrogate consent on behalf of the participant was obtained from either parent or guardian. The participants’ name, address details or any other personal identifier was not collected. Only age, gender and Hgb estimates was captured using unique system generated alphanumeric IDs and used for analysis. Needful permission was obtained from the competent authorities at the health facilities prior to the assessments.

## Results

### Study profile (Table 1)

Hgb assessments were conducted on individuals across 5 sites: Bhubaneshwar (n = 1114) and Solan (n = 111) for EzeCheck versus Hemato-analyzer, Mashobra (n = 92) and Shimla (n = 442) for EzeCheck versus Sahli’s method, and Ulhasnagar (n = 232) for EzeCheck versus Hemato-analyzer versus Hemocue. A total of 1991 individuals were, thus, tested in the age range of 4 to 90 years: 16 children (10 girls and 6 boys) aged 4–11 years, 87 adolescents (39 girls and 48 boys) aged 12–18 years, and 1299 women and 589 men aged ≥19 years.

### Diagnostic performance of EzeCheck with the gold standard (Hematology Analyser)

As per the Haemato Analyzer cut-offs, the prevalence of anemia in the 1991 samples tested was about 46.5%. For detection of anaemia, the Sn and Sp of EzeCheck was mostly in the range of about 85% to 88% for adult women and men; the device also performed in children (Sn = Sp = 100%) and adolescents (Sn~77%, Sp~93%). The positive and negative likelihood ratios suggested EzeCheck as a moderate-to-good device for detection of anaemia across age groups. EzeCheck also had high accuracy (92.8% to 94.9%) as estimated from the area under the ROC curve. Overall, the Sn was 85.1%, Sp = 86.9%, PPV = 85.0, NPV = 87.0%.

If EzeCheck was to be used as a screener tool for dichotomizing individuals into ‘anaemia’ / ‘no anaemia’ with hemato-analyzer cut-offs (as described above), then from the ROC analyses, we identified that the optimal cut-off reading of EzeCheck to detect anaemia was 8.9 gm/dl for children aged 4–11 years (Sn = Sp = Accuracy = 100%), 8.1 gm/ dl for adolescents aged 12–18 years (Sn = 91%, Sp = 82%, Accuracy = 87%), 8.4 gm/ dl for women aged ≥19 years (Sn = 84%, Sp = 87%, Accuracy = 86%), and 7.7 gm/dl for men aged ≥19 years (Sn = 88%, Sp = 85%, Accuracy = 87%).

**Table 1:** Diagnostic performance of EzeCheck with Sysmex XN 1000 Hemato-analyzer as gold standard.

Profile	n	Sn (%) (95% CI)	Sp (%) (95% CI)	PPV (%) (95% CI)	NPV (%) (95% CI)	LR+	LR-	Accuracy (%)
Children aged 4–11 years*	15	100 (100–100)	100 (100–100)	100 (100–100)	100 (100–100)	∞	0	100
Adolescents aged 12–18 years	79	77.27 (68.03–86.51)	92.98 (87.35–98.62)	80.95 (72.29–89.61)	91.38 (85.19–97.57)	11.01	0.24	94.90
Women aged ≥ 19 years	824	84.36 (81.88–86.84)	87.46 (85.2–89.72)	91.42 (89.5–93.33)	77.93 (75.1–80.76)	6.73	0.18	92.80
Men aged ≥19 years	539	88.28 (85.56–90.99)	85.28 (82.29–88.27)	68.82 (64.91–72.73)	95.18 (93.38–96.99)	6.00	0.14	94.71
Overall	1457	85.10 (83.27–86.3)	86.91 (85.17–88.64)	84.98 (83.14–86.81)	87.02 (85.29–88.74)	6.49	0.17	97.70

Sn: Sensitivity; Sp: Specificity; CI: Confidence Interval; PPV: Positive Predictive Value; NPV: Negative Predictive Value; LR+: Positive Likelihood Ratio; LR-: Negative Likelihood Ratio; Accuracy: Area under the ROC curve; ∞: Infinite

*We had just 15 children who had been assessed with both EzeCheck and Hematolyzer. All these children were classified with absolute concordance between the two methods.

### Agreement of EzeCheck with Hemato-analyzer

**Fig 1:**
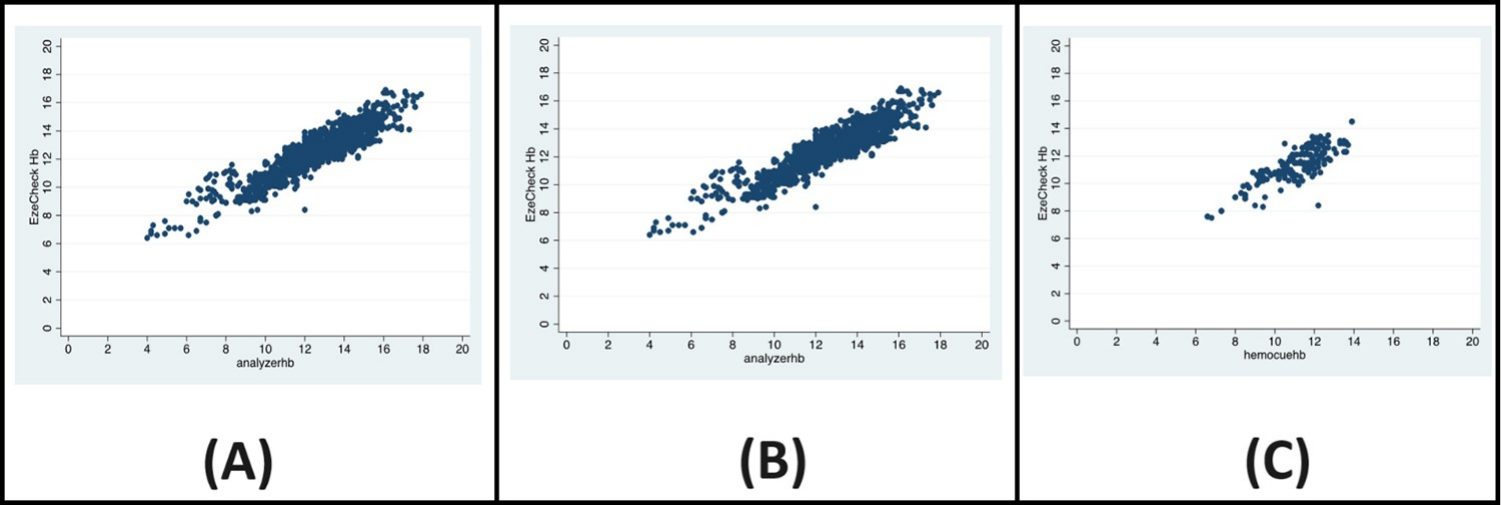
Scatter plots of EzeCheck with Hematolyzer (A), Hemocue (B) and Sahli’s Method (C).

**Fig 2:**
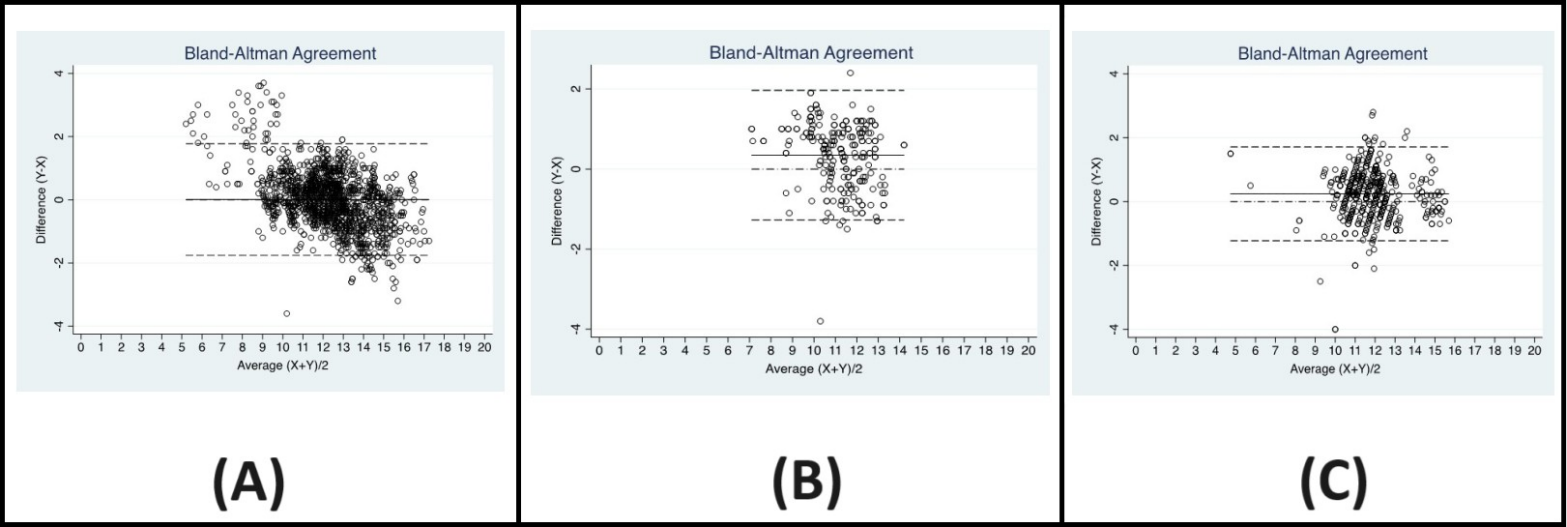
Bland-Altman plots of EzeCheck with Hematolyzer (A), Hemocue (B) and Sahli’s Method (C).

Agreement is usually checked between candidate tests if neither is a ‘gold standard’. Nevertheless, we investigated the agreement between EzeCheck and Sysmex XN 1000 hemato-analyzer considering that the latter may not be an acceptable gold standard for some readers. We noted that there was no statistically significant difference between the readings of EzeCheck and Hemato-analyzer (paired t-test p value = 0.700) and the two correlated well (Fig 1). The *sbsc* between EzeCheck and the Sysmex XN 1000 hemato-analyzer was 0.953 (‘substantial’). EzeCheck and Hemato-analyzer showed high concordance (Lin’s r_ccc_ = 0.89) with high precision (0.91) and accuracy (0.98) (Table 2). The Passing-Bablok regression analysis (Table 3) showed that there was statistically significant constant and proportional bias along with random errors for EzeCheck when compared with the hemato-analyzer. This was, however, negated by the Bland and Altman analysis (Table 3) wherein the bias’s limits of agreement included the line of equality. The Bland and Altman plot (Fig 2) showed that even as the bias was close to zero, it ranged from -1.76 to +1.78 with readings showing consistent agreement majorly around the average Hgb range of 10 gm/dl and above. We could infer that EzeCheck performed in good alignment with hemato-analyzer if the average Hgb was relatively high, and was likely to over-estimate Hgb levels if the average Hgb level was in the lower ranges.

**Table 2:** Comparison and correlation of EzeCheck with Sysmex XN 1000 Hematology Analyzer, Hemocue and Sahli’s Method.

Methods compared	n	Difference of mean (p-value from paired t-test)	Concordance correlation analysis		
			Lin’s r_ccc_ (95% CI)	Precision (ρ)	Accuracy (C_b_)
**EzeCheck/ Hematology Analyzer**	1457	0.700	0.89 (0.881–0.899)	0.911	0.977
**EzeCheck/ Hemocue**	232	<0.001	0.785 (0.738–0.832)	0.819	0.959
**EzeCheck/ Sahli’s Method**	532	<0.001	0.839 (0.814–0.864)	0.852	0.984

**Table 3:** Intercept, slope and residual standard deviation of Passing-Bablok regression as well as bias, lower and upper limit of agreement from the Bland-Altman plots and their 95% confidence intervals (95% CI) from the comparisons of EzeCheck with Sysmex XN 1000 Hematology Analyzer, Hemocue and Sahli’s Method.

Methods compared	Passing-Bablok regression			Bland-Altman plot		
	Intercept (95% CI)	Slope (95% CI)	RSD mean% (SD %)	Bias (95% CI)	LoA (95% CI)	UoA (95% CI)
**EzeCheck/ Hematology Analyzer**	2.171 (1.950 to 2.460)*	0.822 (0.800 to 0.842)*	1.11 (9.30)	0.009 (-0.037 to 0.055)	-1.758 (-1.837 to -1.678)	1.776 (1.696 to 1.855)
**EzeCheck/ Hemocue**	2.064 (1.285 to 2.747)*	0.857 (0.789 to 0.928)*	3.72 (7.73)	0.346 (0.240 to 0.453)*	-1.272 (-1.458 to -1.087)	1.966 (1.780 to 2.151)
**EzeCheck/ Sahli’s Method**	0.300 (0.300 to 1.146)*	0.100 (0.923 to 1.000)	2.39 (6.88)	0.243 (0.179 to 0.307)*	-1.226 (-1.337 to -1.115)	1.712 (1.601 to 1.822)

*statistically significant RSD: Residual standard deviation; CI: Confidence Interval; LOA: Lower limit of agreement; UOA: Upper limit of agreement

### Agreement of EzeCheck with Hemocue and Sahli’s method

The readings of EzeCheck were different from that with Hemocue and Sahli’s method (paired t-test p value<0.001). The *sbsc* of EzeCheck was 0.900 (‘moderate’) with Hemocue and 0.920 (‘moderate’) with Sahli’s method. EzeCheck when compared to each of the latter two i.e. Hemocue and Sahli’s methods, however, showed good concordance (Lin’s r_ccc_ = 0.785 and 0.839 respectively), precision (0.819 and 0.852 respectively) and accuracy (0.959 and 0.984) on concordance correlation analysis. (Table 2)

The Passing-Bablok regression analysis (Table 3) showed that there was statistically significant constant and proportional bias for EzeCheck with respect to Hemocue. There was statistically significant constant bias when EzeCheck was compared to Sahli’s method but no proportional bias. Bland-Altman plots (Fig 2) indicated bias between EzeCheck and the other two tests.

## Discussion

Different non-invasive devices are available nowadays with varied technological aspects. Although the approach remains the same for all the devices i.e., non-invasive method for determining the haemoglobin levels but the accuracy aspect for all those available devices differs greatly. In case of Masimo Radical 7, accuracy with 0.2g/dL of standard deviation is observed whereas in case of Pronto-7 and Orsense NBM 200, accuracy with 1.1g/dL of S.D and 0.86g/dL of S.D respectively is observed in comparison with the laboratory values[21].

Similarly, in the case of EzeCheck device, accuracy with 0.24% biasing was observed at 95% CI when the device was compared with the traditional lab method for haemoglobin detection. This paper compared the diagnostic performance of the non-invasive point-of-care device EzeCheck with three different Hgb assessment methods i.e., the whole blood hemato-analyzer (Sysmex XN 1000) as the gold standard, traditional Sahli’s method and the portable invasive (finger-prick) hemoglobinometer HemoCue (Hb-301). We found that EzeCheck performed well across age and gender categories with convincing sensitivity, specificity, concordance and accuracy. On Bland-Altman Analysis [22] comparing EzeCheck with Hemato-analyzer, the line of equality (zero) lay within the 95% confidence interval for systematic bias and thus, the systematic bias was not significant. When compared with hemato-analyzer, spot check hemoglobin monitors have been shown to have minimal bias ±standard deviation of −0.1 ± 1.1 gm/dL and accuracy similar to invasive phlebotomy [23]; this was similar to that of EzeCheck which showed a bias of 0.009 ± 0.9 gm/dl. The EzeCheck device showed significant systematic bias when compared to the other two methods. We, however, had a stringent margin (±1 gm/dl) for the level of agreement (LOA) on Bland-Altman analysis for assessing total bias (systematic and random bias). The 95% LOA limits ranged within 2.0 thus, indicating an otherwise acceptable margin for bias had the pre-set allowable clinical error been set at 2.0 gm/dl instead of 1.0 gm/ dl [24]. Previous researchers have suggested that methods having a difference of beyond 2.0 gm/dl were unacceptable and those within 2.0 gm/dl could be considered as potential replacement candidates for hemoglobin assessment methods [25]. There have been reports suggesting that non-invasive hemoglobinometers using spectrophotometry techniques have good concordance with automatic hemato-analyzer and with invasive bed-side methods [26].

Since we attempted to conduct the assessments in real world (implementation) settings and within routine practice protocols, we did not control the participant selection. However, we acknowledge that an analysis of the required sample size would add to our conviction with the findings. Hence, we used the Everald’s equation for power calculation in diagnostic tests [27]. Assuming a sensitivity and specificity of 85% each and prevalence of anemia of about 50% (as per hematolyzer cut-offs, the prevalence of anemia in the participants was 46.5%), the overall sample size was adequate for estimating validity of EzeCheck against hematolyzer (N = 1991; relative precision ~2.64%); it was also adequate for stratified analysis for adult women (N = 824; relative precision ~ 4%), adult men (N = 539; relative precision ~5%), and adolescent children (N = 79; relative precision 13.5%).

Despite its strengths, there are some limitations in our report. The present assessment had only 16 participants in the 4-to-11-year age band. The presence of cut marks or sweat or stains like mehndi/tar/turmeric etc., on the fingertip will provide erroneous results as it obstructs the passage of light. Extreme cold hands (below 15°C) may interfere with the proper blood flow to the fingertip as the blood vessels constrict in extreme low temperature, thereby affecting the results. In the present paper, the data for the device validation was conducted in four states but all in the rainy season. Since the findings of hemoglobinometers is shown to get affected by temperatures, especially extreme temperatures, the device needs to be tested across variegated temperatures and altitudes. There is also the need for qualitative exploration with the patients and users of EzeCheck to obtain in-depth insights into the convenience and barriers of the usability of the device in the real field setting. This in turn would help to understand acceptability and amplify uptake of the device at the community level. Based on the perceived benefits (non-invasive method, no/ minimal need for training, instant reporting, automatic data archival, and low equipment cost), EzeCheck appears to be cost-effective; however, economic modelling exercises are mandated to prove this. We reckon that the real-world data context of this analysis adds to the robustness of the inferences drawn about EzeCheck’s performance.

We conclude that for operational ease and taking cues from the ROC analysis, if EzeCheck was calibrated with a cut-off value set at 8.0 to detect anemia (for values > = 8) across age and gender categories, then it would perform with a Sn ≅ Sp ≅ Accuracy ≅ 85.0% for detecting anaemia as compared to the hemato-analyzer estimates. Further, for countries with high prevalence of anemia where universal screening may be mandated, the positive predictive value of EzeCheck will be even more favourable. The likelihood ratios also indicated that the device had moderate-to-good utility. There are certain situations where EzeCheck’s readings should be interpret with caution e.g., extremely low values of hemoglobin e.g., patients in shock or with compromised peripheral perfusion; non-invasive spot hemoglobin monitors tend to underperform in such situations [28]. Nevertheless, EzeCheck holds the potential to be used as an effective screening tool for anemia at the frontline across age and gender categories. It is also well-poised for embedment into program and out-patient health care settings in resource constrained contexts as a spot-check hemoglobinometer.
